# Editorial: Bisphenols and Male Reproductive Health

**DOI:** 10.3389/fendo.2020.597609

**Published:** 2020-10-08

**Authors:** Arcangelo Barbonetti, Settimio D'Andrea, Nicola Bernabò, David H. Volle

**Affiliations:** ^1^Andrology Unit, Department of Life, Health and Environmental Sciences, University of L'Aquila, L'Aquila, Italy; ^2^Faculty of Bioscience and Technology for Food, Agriculture and Environment, University of Teramo, Teramo, Italy; ^3^Inserm U1103, CNRS UMR6293–Université Clermont Auvergne, Laboratory Genetic, Reproduction & Development, Clermont-Ferrand, France

**Keywords:** bisphenol, endocrine disruptors, male fertility, spermatozoa, spermatogenesis

Bisphenols are organic industrial chemicals, widely used in the manufacture of plastic articles such as polyvinylchloride (PVC), polycarbonate plastics, and epoxy resins. Currently, bisphenol A (BPA), which represents the first-choice plasticizer due to its cross-linking properties, is produced and used in the highest volumes worldwide. Leaching of BPA monomers from inner coating of food and drink containers, especially with repeated use and following exposure to high temperature, largely accounts for the widespread human exposure to BPA by oral ingestion. However, equally important alternative non-dietary routes of absorption, including inhalation and transdermal route, have been demonstrated. Accordingly, in the National Health and Nutrition Examination Survey (NHANES), over 90% of the study population exhibited measurable urinary concentrations of BPA (1).

The ubiquitous presence and environmental persistence of BPA, along with its reputation of being an endocrine disruptor, is generating worldwide concerns about the possible links with a spectrum of human health disorders, including infertility. Due to the resultant restrictions in BPA production, the increasing use of BPA analogs is attracting interest to these new compounds, which, however, could share chemical and biological properties similar to BPA.

This special issue provides an overview of more recent clinical and basic insights about the possible impact of bisphenols on male reproductive health and expresses the opinions of experts from different areas of medicine and biology who have expanded the field with their recent discoveries.

Results from preclinical research clarified possible mechanisms by which BPA can interfere with the regulation of spermatogenesis (Castellini et al.; De Toni et al.). A polycyclic phenolic chemical structure, similar to estradiol, confers to BPA estrogenic activities exerting disrupting effects on the feedback regulation of the hypothalamic–pituitary–gonadal axis. The decreased pituitary secretion of luteinizing hormone (LH) and hypostimulation of Leydig cell steroidogenesis results in lower intratesticular levels of testosterone, which plays a pivotal role in fetal development as well as in adulthood maintenance of secondary sexual function and spermatogenesis. In addition, bisphenols can exert direct harmful effects at testicular levels. In *in vitro* studies, BPA promoted mitochondrial dysfunction, apoptosis and DNA damage of Sertoli cells with disruption of the blood-testis barrier integrity (Adegoke et al.). Detrimental reflections on spermatogenesis would be further exacerbated by intratesticular direct and indirect anti-androgenic activities, as BPA interferes with Leydig cell development and expression of steroidogenic enzymes, as well as with androgen receptor signaling (Adegoke et al.; Barbagallo et al.; Castellini et al.; Li et al.). Of note, many of these effects could be shared by several BPA analogs, which display properties of estrogen receptor agonists and androgen receptor antagonists (Li et al.).

Experimental studies also suggest that bisphenols could extend their biological effects on male fertility beyond the disruption of the spermatogenesis regulation. Exposure to BPA has been shown to promote epigenetic modifications in both animal and human cells, resulting in endocrine derangements, microscopic and macroscopic abnormalities of male reproductive system as well as inheritable epigenetic changes involving human reproduction (Cariati et al.). Direct effects of bisphenols on sperm functions have been also reported. In different species, including human (2), the *in vitro* exposure of spermatozoa to BPA induced pro-oxidative and apoptotic mitochondrial dysfunctions, resulting in the loss of sperm motility, viability, and DNA integrity. Furthermore, in human spermatozoa, bisphenols BPG, BPAF, BPC, BADGE, BPB, and BPBP can interfere with physiological signaling of the sperm-specific Ca^2+^ channel CatSper (Rehfeld, Andersson et al.), which is activated by the female sex steroid progesterone and plays a key role in the acquisition of sperm fertilizing ability. However, molecular mechanisms leading to activation of CatSper differ between the species, as BADGE and progesterone failed to induce Ca^2+^ signals in boar spermatozoa (Rehfeld, Mendoza et al.).

Overall, while preclinical research has provided compelling evidence that bisphenols can negatively interfere with male reproduction, clinical studies have produced quite inconclusive results. With the exception of few reports on the relationship of prenatal exposure to BPA with abnormal androgen status and pubertal timing (Hart), the claimed clinical adverse effects of bisphenols on male fertility are largely inferred from conventional semen analysis, which, however, is burdened by a number of limitations (Castellini et al.). To date, any conclusion about the cause–effect relationships is hindered by the cross-sectional design of the studies and the large spontaneous between- and within-subject variability of semen parameters (3). Furthermore, despite the adjustment for possible confounding factors in different studies, other unmeasured confounders could have influenced the associations under investigation. Obviously, the best evidence of an adverse effect of BPA on male fertility would be provided by longitudinal analyses, assessing clinically relevant endpoints, such as natural or medically assisted pregnancies among men either with different exposure degrees or with different clinical conditions (fertile/subfertile).

While this latter represents a real challenge for future research, we would like to express our sincere gratitude to all authors and referees for their contribution to this issue summarizing the multidisciplinary and collaborative efforts which in recent years have helped shed some light on a topic yet to be largely investigated.

## Author Contributions

All authors listed have made a substantial, direct and intellectual contribution to the work, and approved it for publication.

## Conflict of Interest

The authors declare that the research was conducted in the absence of any commercial or financial relationships that could be construed as a potential conflict of interest.
